# MSCs inhibit tumor progression and enhance radiosensitivity of breast cancer cells by down-regulating Stat3 signaling pathway

**DOI:** 10.1038/s41419-018-0949-3

**Published:** 2018-10-08

**Authors:** Ningning He, Yangyang Kong, Xudan Lei, Yang Liu, Jinhan Wang, Chang Xu, Yan Wang, Liqing Du, Kaihua Ji, Qin wang, Zongjin Li, Qiang Liu

**Affiliations:** 1 Tianjin Key Laboratory of Radiation Medicine and Molecular Nuclear Medicine, Department of Radiobiology, Institute of Radiation Medicine of Chinese Academy of Medical Science and Peking Union Medical College, Tianjin, China; 2 School of Medicine, Nankai University, Tianjin, China

## Abstract

The acquisition of radioresistance by breast cancer cells during radiotherapy may lead to cancer recurrence and poor survival. Signal transducer and activator of transcription 3 (Stat3) is activated in breast cancer cells and, therefore, may be an effective target for overcoming therapeutic resistance. Mesenchymal stem cells (MSCs) have been investigated for use in cancer treatment. Here, we investigated the potential of MSC conditioned medium (MSC-CM) in sensitizing breast cancer to radiotherapy. It was found that MSC-CM could inhibit the level of activated Stat3, suppress cancer growth, and exhibit synergetic effects with radiation treatment in vitro and in vivo. Furthermore, MSC-CM reduced the ALDH-positive cancer stem cells (CSCs) population, modulated several potential stem cell markers, and decreased tumor migration, as well as metastasis. These results demonstrate that MSC-CM suppresses breast cancer cells growth and sensitizes cancer cells to radiotherapy through inhibition of the Stat3 signaling pathway, thus, providing a novel strategy for breast cancer therapy by overcoming radioresistance.

## Introduction

Breast cancer is the most common malignancy and is the leading cause of cancer-related deaths in females worldwide^1,2^. Currently, the major clinical therapeutic methods for breast cancer include traditional surgical treatment, chemotherapy, and radiotherapy. Among them, radiotherapy is an important treatment modality to achieve local control and reduce the risk of recurrence. However, its curative effect is sometimes limited by radioresistance of cancer cells. Recently, the regulation of tumour radiosensitivity has attracted much attention, and identification of novel radiosensitizing agents that can increase the radiosensitivity of breast cancer has become an area of interest for radiation oncology investigators.

Several studies have shown that mesenchymal stem cells (MSCs) could be used to treat and enhance the radiosensitivity of cancer cells^3,4^. MSCs are multipotent cells that reside in various tissues and have the potential of multidirectional differentiation, which allows these cells to differentiate into multiple mesodermal cell lineages^5–8^. MSCs have been isolated from many different tissues, including bone marrow, adipose tissue, umbilical cord blood, peripheral blood, and skeletal muscle^9,10^ and are a promising source for cell therapy in regenerative medicine. While several studies have demonstrated that MSCs contribute to tumour progression and metastasis^11,12^, other reports have shown that MSCs could suppress tumour growth^13,14^. The different effects of MSCs on tumour growth depend on a variety of factors, including the type and origin of MSCs, the tumour models, and the dose and time of administration of cell treatments^15^. Therefore, it is necessary to explore the potential mechanisms of MSC-induced tumour inhibitory effects in breast cancer cells.

Signal transducer and activator of transcription 3 (Stat3) played a vital role in tumourigenesis^16–18^. An early research of human breast cancer cell lines demonstrated that Stat3 was activated in five of the nine cell lines^19,20^. Stat3 activation is found in all classes of breast cancers, but is most often associated with triple negative breast tumors. The Stat3 signaling pathway was recently reported to contribute to tumour progression and the survival of breast cancer-derived stem cells. Some studies have shown that the Stat3 signaling pathway is required for growth of CD44^+^CD24^–^ stem cell–like breast cancer cells^21^, such as several basal-like breast cancer cells (MDA-MB-231, BT-549, HCC1937, Hs 578T, MDA-MB-468, and SUM159PT ), not expressed in luminal breast cancer cell lines (BT-474, MCF7, MDA-MB-453, SK-BR-3, T-47D, and ZR-75-1)^22^. However, whether the tumour inhibitory effect of MSCs is mediated by the Stat3 signaling pathway is unclear.

In this study, we used MSC-conditioned medium (MSC-CM) combined with radiation treatment and an imaging approach to explore how the aggressive breast cancer cells (MDA-MB-231) respond to the combination treatment and to investigate the possible underlying mechanisms. Our results indicated that MSC-CM reduces the growth of MDA-MB-231 cells and sensitises the cancer cells to radiation therapy through inhibition of Stat3 activation. This work identifies Stat3 as a potential therapeutic target that may radiosensitise cells prior to conventional radiation therapy and provides a basis for the clinical application of radiation combined with MSC therapy, thus suggesting a more effective treatment for breast cancer patients.

## Results

### Construction of optical imaging tumour cells

**Fig. 1:**
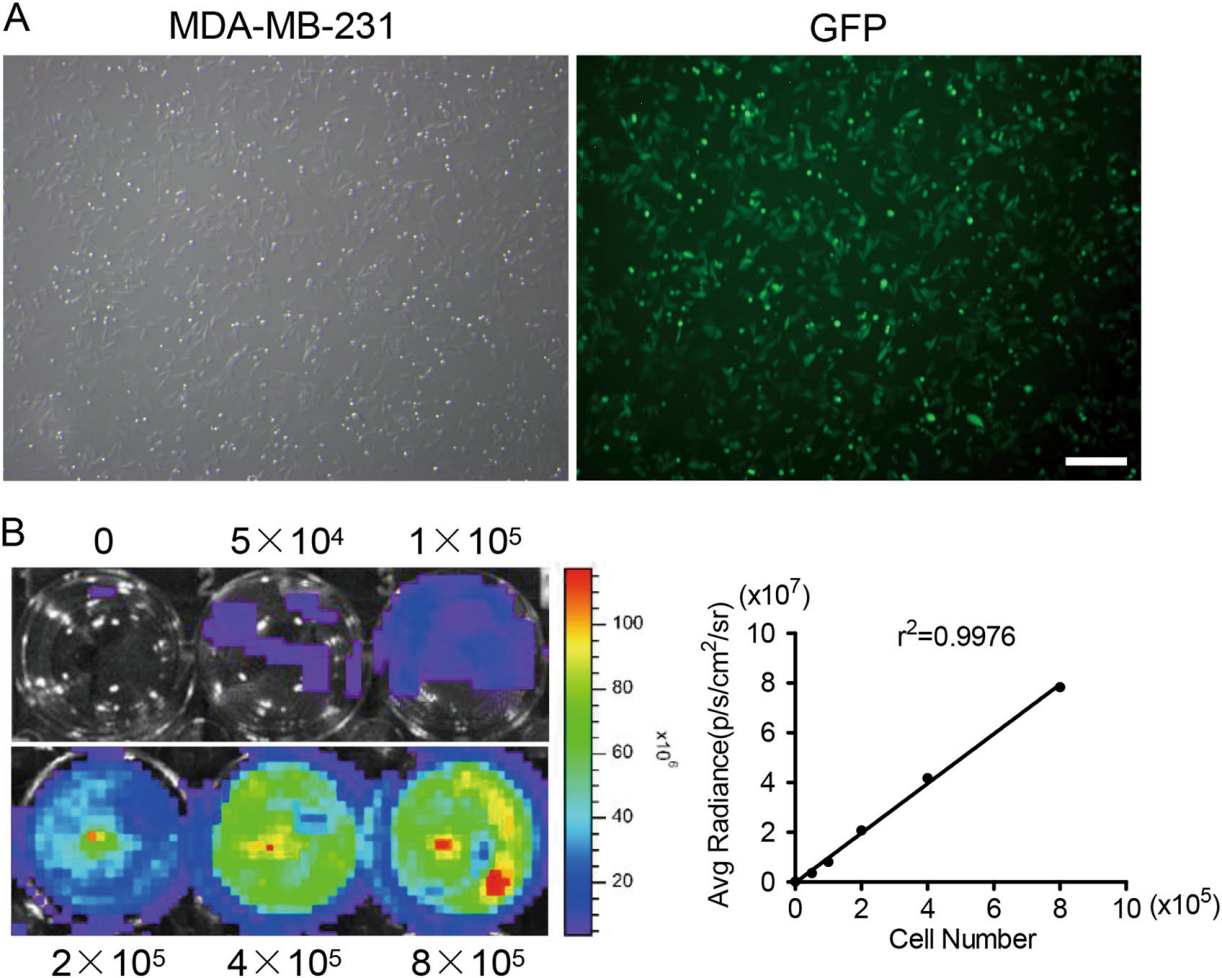
Transduction of MDA-MB-231 cells with Fluc/GFP and pStat3/Rluc reporter genes. (**a**) Transduced MDA-MB-231 cells were strongly positive for eGFP as shown by fluorescence microscopy. (**b**) Ex vivo imaging analysis of stably transduced MDA-MB-231 cells showed a strong correlation between cell numbers and Fluc reporter gene activity. The scale bar represents 50 μm

To evaluate the effect of the MSCs on cancer cells and track the transplanted cancer cells in vivo using imaging analysis, we constructed double imaging MDA-MB-231 cells (Fluc/GFP-pStat3/Rluc) with Fluc and eGFP reporter genes drived by a ubiquitin promoter, Rluc reporter gene drived by a seven-repeat Stat3-binding sequence (enhancer) and minimal TA (promoter) in response to the activated Stat3. The fluorescence images showed that the expression of eGFP was robust in MDA-MB-231 cells (Fig. 1a). FACS analysis indicated that GFP was expressed in >95% of cells after sorting (data not shown). A strong correlation (r^2^ = 0.9976) between the cell number and firefly signal intensity was observed in vitro using the Xenogen IVIS system, which quantified tumour cells by analysing firefly signal intensity (Fig. 1b). The Rluc expression was controlled by Stat3 activation. Once activated, the phosphorylated Stat3 underwent dimerization and entered the nucleus to bind the seven-repeat response elements inducing the expression of Rluc. When the cells were administrated with coelenterazine, photon signals could be detected. The signaling intensity of Rluc therefore indicated the activation of Stat3 signaling in cancer cells.

### Tumour suppressor activity of MSC-CM and its combination with radiotherapy

**Fig. 2:**
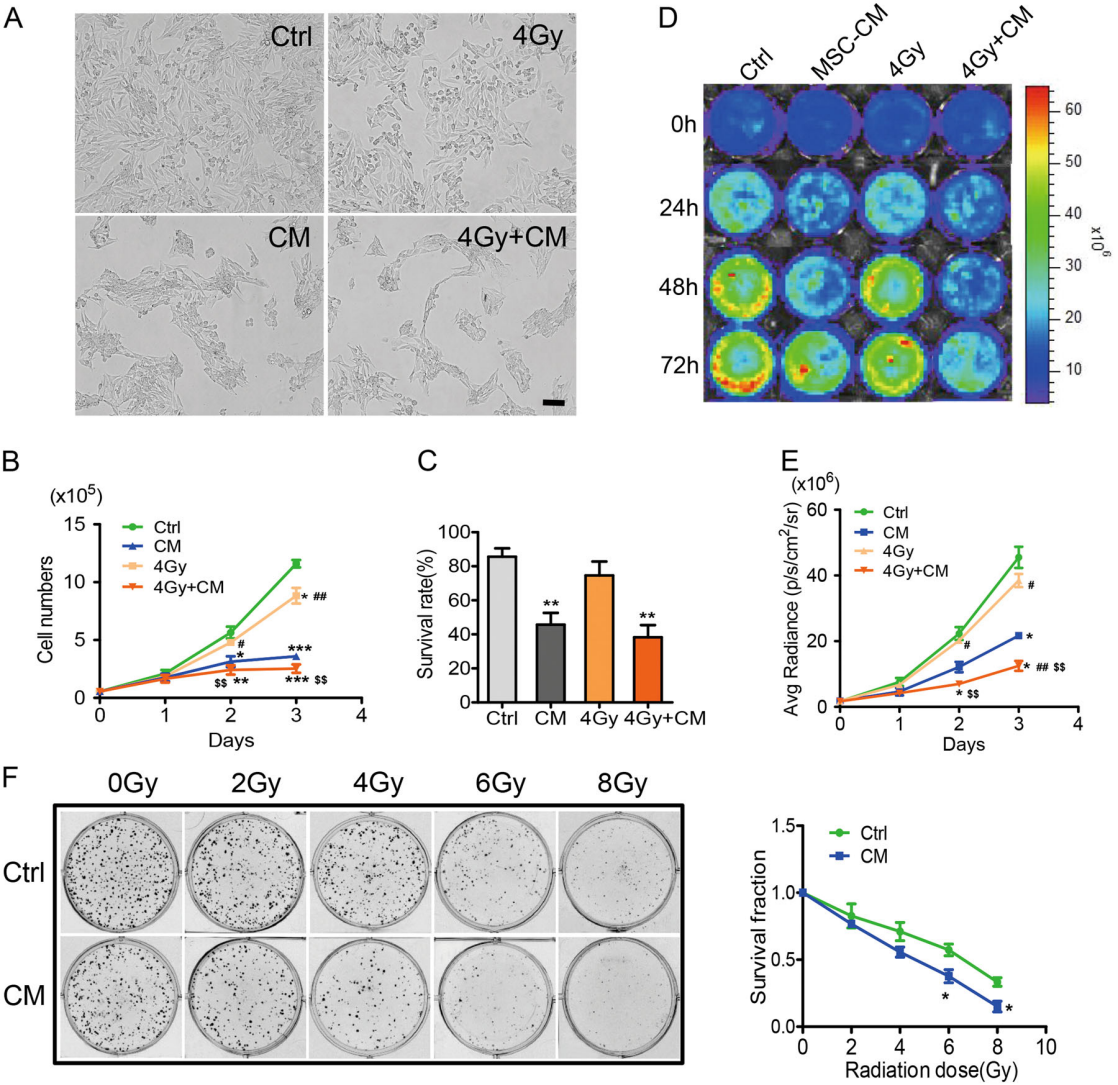
Effect of MSC-CM on the proliferation of aggressive breast cancer cells. (**a**) Microscopic analysis showed morphological changes in cells treated with MSC-CM, 4 Gy radiation and MSC-CM combined with 4 Gy radiation. The scale bar represents 100 μm. (**b**) Growth curves made by counting cell numbers every day revealed a decreased proliferation rate of cells treated by MSC-CM and the MSC-CM combination with 4 Gy radiation. *P < 0.05 vs Ctrl, **P < 0.01 vs Ctrl, ***P < 0.001 vs Ctrl, ^#^P < 0.05 vs MSC-CM, ^##^P < 0.01 vs MSC-CM, ^$$^P < 0.01 vs 4 Gy, n = 3. (**c**) Trypan blue cell viability assays showed a low survival rate. **P < 0.01 compared to controls, n = 3. (**d**) Fluc imaging showed decreasing bioluminescence signals at 24, 48, and 72 h in the MSC-CM-treated and combination groups. (**e**) Quantitative analysis of Fluc imaging signals. The signal activity showed the suppressed growth in the combination group. *P < 0.05 vs Ctrl, ^#^P < 0.05 vs MSC-CM, ^##^P < 0.01 vs MSC-CM, ^$$^P < 0.01 vs 4 Gy, n = 3. (**f**) MDA-MB-231 cells treated with control medium and MSC-CM were exposed to ionizing radiation. Cell growth was analysed by colony formation assays as described in the Materials and Methods. Reported values are the mean±SD for three separate experiments

To investigate the effect of MSCs on breast cancer cells, we treated MDA-MB-231 cells with MSC-CM firstly. The cell proliferation and survival were decreased (Figure S1). Then we treated MDA-MB-231 cells with MSC-CM, radiation, and MSC-CM in combination with radiation to examine the effect of MSC-CM on tumour radiosensitivity. MDA-MB-231 cells in the MSC-CM-treated and combination groups lost their normal spindle shape and became thinner and longer after 48 h of treatment, the number of cells was sharply reduced (Fig. 2a). Furthermore, the proliferation rate of tumour cells in the MSC-CM-treated and combination groups grew slowly compared with the control groups. As shown in Fig. 2b, MSC-CM could inhibit the growth of tumour cells, however, there was no obvious difference between the control and the only radiation group. The imaging assay showed decreased Fluc activity in cells treated with MSC-CM and the combination groups (Fig. 2d). Quantitative analysis demonstrated that the Fluc signal intensity of tumour cells in the combination group was less than the control groups (Fig. 2e). The cell survival assay was performed using Trypan blue staining, which showed a decrease in the MSC-CM and combination groups (Fig. 2c). To determine the effect of MSCs on tumour cells radioresistance in vitro, we performed colony formation assays. A clonogenic survival assay showed that MSC-CM increased cell radiosensitivity compared with that of the control group (Fig. 2f).

### MSC-CM promotes apoptosis and inhibits the cell cycle of breast cancer cells

**Fig. 3:**
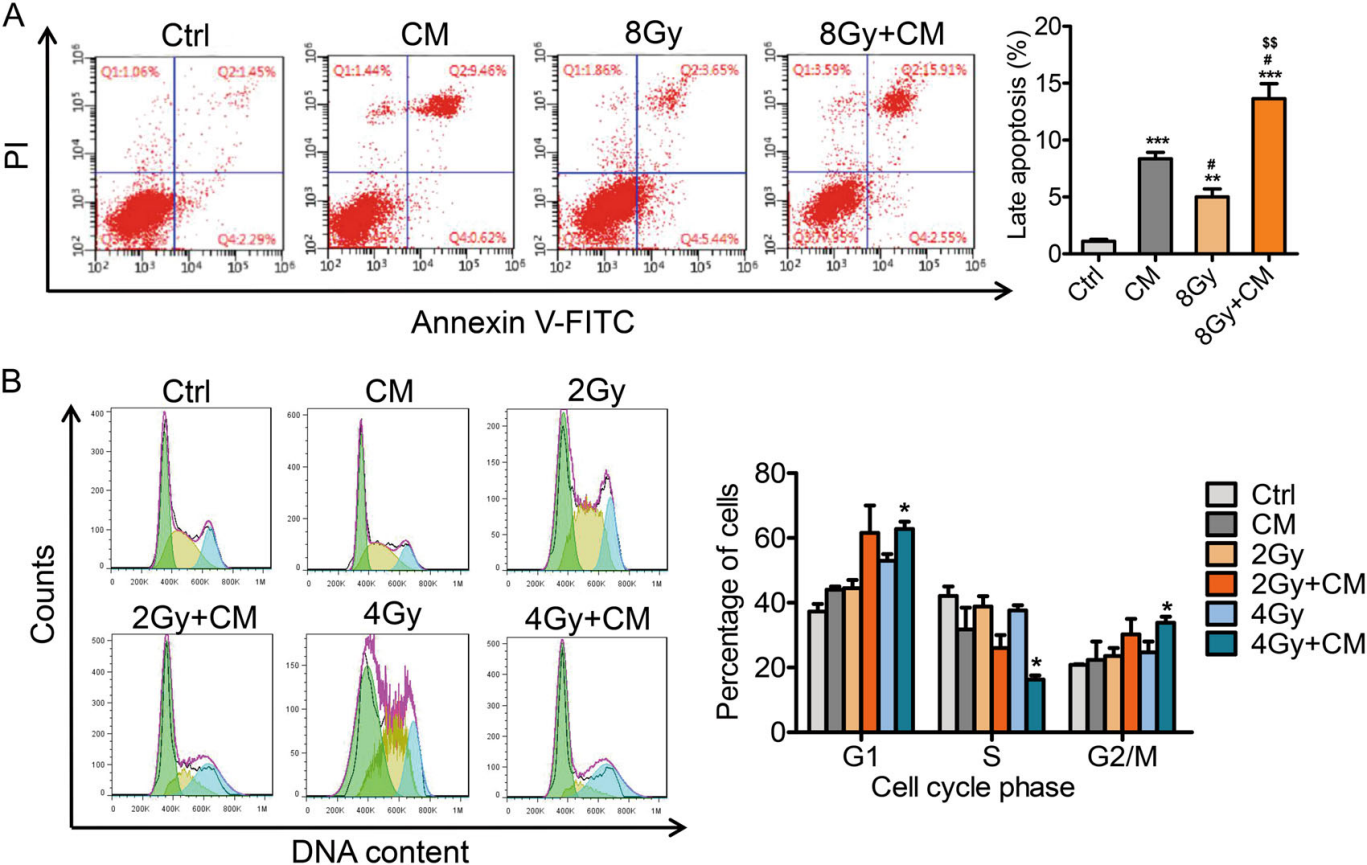
Effect of the MSC-CM combination with radiotherapy on cell apoptosis and cell cycle progression in MDA-MB-231 cells. (**a**) MDA-MB-231 cells were treated with control medium and MSC-CM and then exposed to 8 Gy ionizing irradiation. The apoptosis of cells was assessed by FACS after 48 h of irradiation. (**b**) The cells were treated with control medium and MSC-CM and then at 24 h after exposed to 2 Gy or 4 Gy radiation, stained with PI and analysed using FACS. The proportion of DNA in the different cell phases was analysed. Data are shown as the mean±SD (error bars) from three independent experiments. *P < 0.05 vs Ctrl, **P < 0.01 vs Ctrl, ^#^P < 0.05 vs MSC-CM, ^##^P < 0.01 vs MSC-CM, ^$$^P < 0.01 vs 8 Gy, n = 3

Irradiation primarily leads to the production of double-stranded DNA breaks (DSBs), which result in cell apoptosis. Radiation leads to cell apoptosis, which could be understood as radiosensitivity^23,24^. Here, we evaluated the apoptosis of tumour cells affected by MSC-CM. Flow cytometry apoptosis assays showed that MSC-CM increased apoptosis of tumour cells compared with that of the control groups, especially in cells exposed to 8 Gy radiation (Fig. 3a). Previous studies showed that most radioresistant cells were arrested in S phase, with fewer cells in G2-M phase, after irradiation compared with radiosensitive cells^25^. Accordingly, we further studied the cell cycle changes in response to radiation and MSC-CM using flow cytometry. As shown in Fig. 3b, no difference was observed in the G0-G1, S and G2-M phases in the control, CM or radiation only groups; however, compared with these groups, the combination group had fewer cells in S phase and more cells in G2-M phase, suggesting that combined treatment of MSC-CM with radiation could lead to the cell cycle block at G2-M phase, which is consistent with the typical radiosensitive phenotype. Taken together, these results demonstrated that MSC-CM could sensitise MDA-MB-231 cells to irradiation in vitro.

### MSC-CM inhibits the Stat3 pathway in breast cancer cells

Recent studies demonstrated that Stat3 plays critical roles in the initiation and progression of breast cancer^19^. The most common cause of disease relapse and radioresistance in breast cancer is the presence of stem cell-like cells (or CSCs) in tumour tissues^26^. Multiple signaling pathways, including the Stat3 signaling pathway, help maintain stem cell programmes in normal cells as well as in cancer cells^27–29^.

**Fig. 4:**
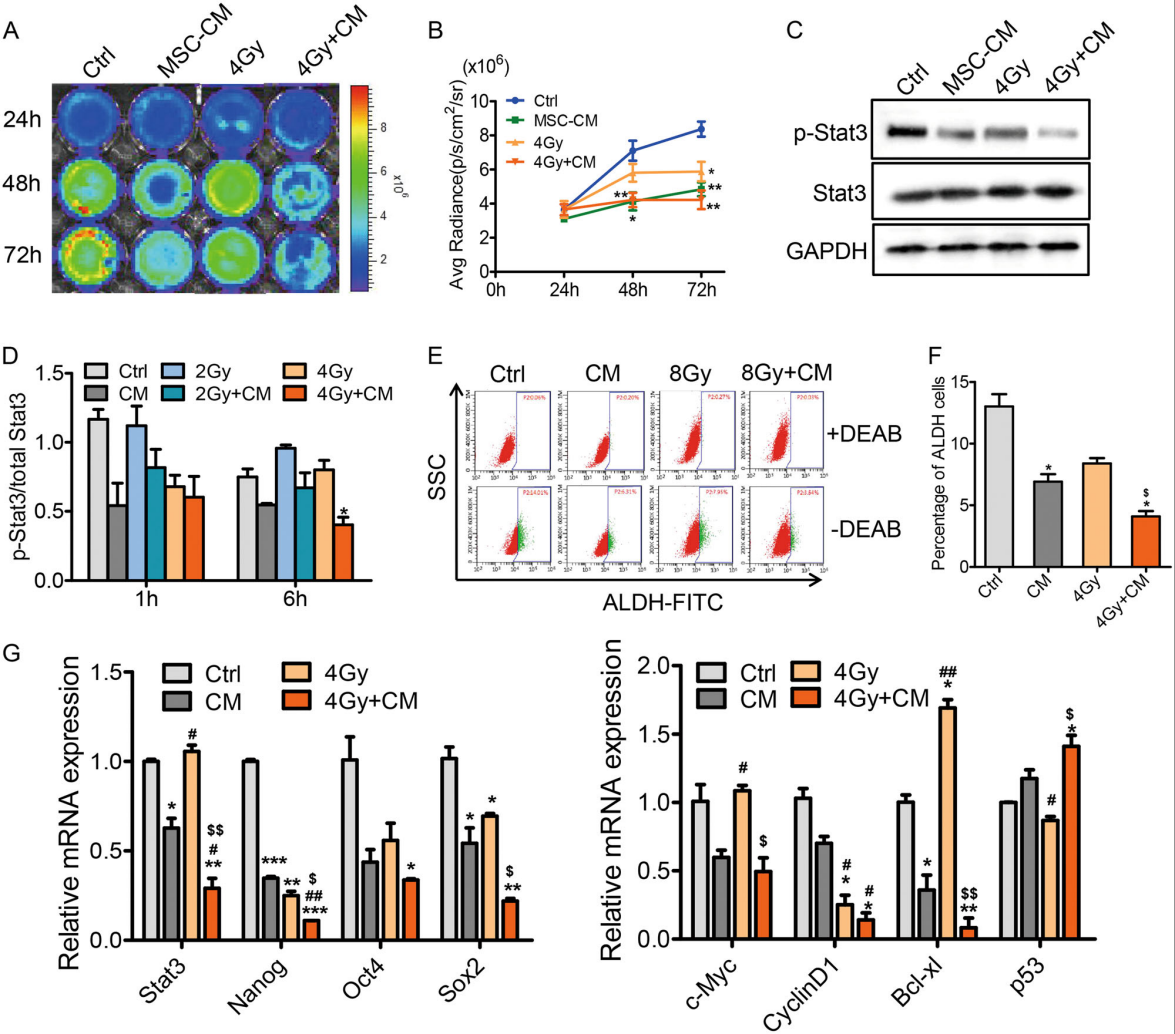
MSC-CM inhibited the Stat3 signaling pathway and decreased self-renewal of breast cancer stem cells in MDA-MB-231 cells. (**a**) Renilla luciferase (Rluc) imaging of activated Stat3 in vitro. (**b**) Quantitative analysis of imaging signals. The signal activity showed the suppression in the combination group. *P < 0.05 vs Ctrl, **P < 0.01 vs Ctrl, n = 3. (**c**) The expression of Stat3, phosphorylated (Try-705) Stat3 measured with Western blotting. (**d**) Quantification of Stat3 phosphorylation; the phospho-Try705 Stat3 and total Stat3 protein levels in cells were quantitatively determined using a target specific primary antibody and HRP-conjugated secondary antibody detection agent, and the crystal violet provided whole cell staining that was used for cell number counts. The OD value was normalized by cell counts. *P < 0.05 vs Ctrl, n = 3. (**e**) Aldehyde dehydrogenase (ALDH) activities in MDA-MB-231 cells were assessed utilizing the ALDEFLUOR assay. Cells were incubated with 1.5 μl of activated ALDEFLUOR substrate at 1 × 10^6^ cells per ml. Diethylaminobenzaldehyde (DEAB) was used to establish the baseline fluorescence of these cells and to define the ALDEFLUOR-positive region. Incubation of cells with ALDEFLUOR substrate in the absence of DEAB induces a shift in BAAA fluorescence, defining the ALDEFLUOR-positive population. (**f**) Quantitative analysis of ALDEFLUOR-positive cells. The number of ALDEFLUOR-positive cells was reduced after treatment with MSC-CM, especially when combined with the irradiation treatment. *P < 0.05 vs Ctrl, ^$^*P* < 0.05 vs 4 Gy, n = 3. (**g**) PCR analysis of Stat3 signalling pathway-related gene expression. *P < 0.05 vs Ctrl, **P < 0.01 vs Ctrl, ***P < 0.001 vs Ctrl, ^#^*P* < 0.05 vs MSC-CM, ^##^*P* < 0.01 vs MSC-CM, ^$^*P* < 0.05 vs 4 Gy, ^$$^*P* < 0.01 vs 4 Gy, n = 3

To explore the activation of the Stat3 signaling pathway in MDA-MB-231 cells in different groups, we performed Rluc imaging, which showed the activated Stat3 level. After 48 h and 72 h treated with MSC-CM, the Rluc activity was significantly decreased in the MSC-CM and the combination groups as shown by the Renilla signal intensity (Fig. 4a, b). Stat3 phosphorylated at Try-705 was substantially decreased in cells treated with MSC-CM and MSC-CM combined with radiation groups as shown by western blot and cell-based ELISA (Fig. 4c, d). Then, we studied the expression of stemness genes (Sox2, Oct4, Nanog, and c-Myc) and Stat3 signaling pathway-related genes (cyclin D1, Bcl-xl, p53) affected by MSC-CM; these genes were substantially down-regulated in the combination group compared with the control groups (Fig. 4g). These results indicated that MSC-CM suppressed Stat3 signaling activation. As MSC-CM exhibited an inhibitory effect on Stat3 activation, we further examined its anti-CSC properties. The CSCs in breast cancer cells were assessed using ALDEFLUOR and Mammosphere formation assays. ALDEFLUOR-positive cells (Fig. 4e, f) and the mammosphere formation efficiency (Figure S3) were reduced after treatment with MSC-CM for 48 h, especially when combined with the irradiation treatment.

To further detect MSC-CM could sensitize cancer cells to radiotherapy via inhibiting Stat3 signaling pathway, we used Stat3 inhibitor Stattic to inhibit the Stat3 signaling pathway and then analyzed the radiosensitivity of cancer cells affected by MSC-CM. The cells survival, epithelial-mesenchymal transition, metastasis, and angiogenesis were inhibited in MSC-CM combination with radiation group after treated with 5 uM Stattic (Figure S4).

### Effects of MSC-CM on breast cancer cells migration, metastasis, and angiogenesis

Epithelial-to-mesenchymal transition (EMT) plays an important role in metastasis and invasiveness of cancer cells and is associated with poor clinical outcome in lots of cancers^30–32^. EMT is an important process involved in progression of cancers, and previous studies have demonstrated that Stat3 inhibition leads to EMT characterised by up-regulation of epithelial cell-specific proteins (E-cadherin), and by down-regulation of the mesenchymal cell-specific protein N-cadherin^33^. The Stat3 pathway is tightly associated with tumour invasion, metastasis, and angiogenesis in cancers^34–36^.

**Fig. 5:**
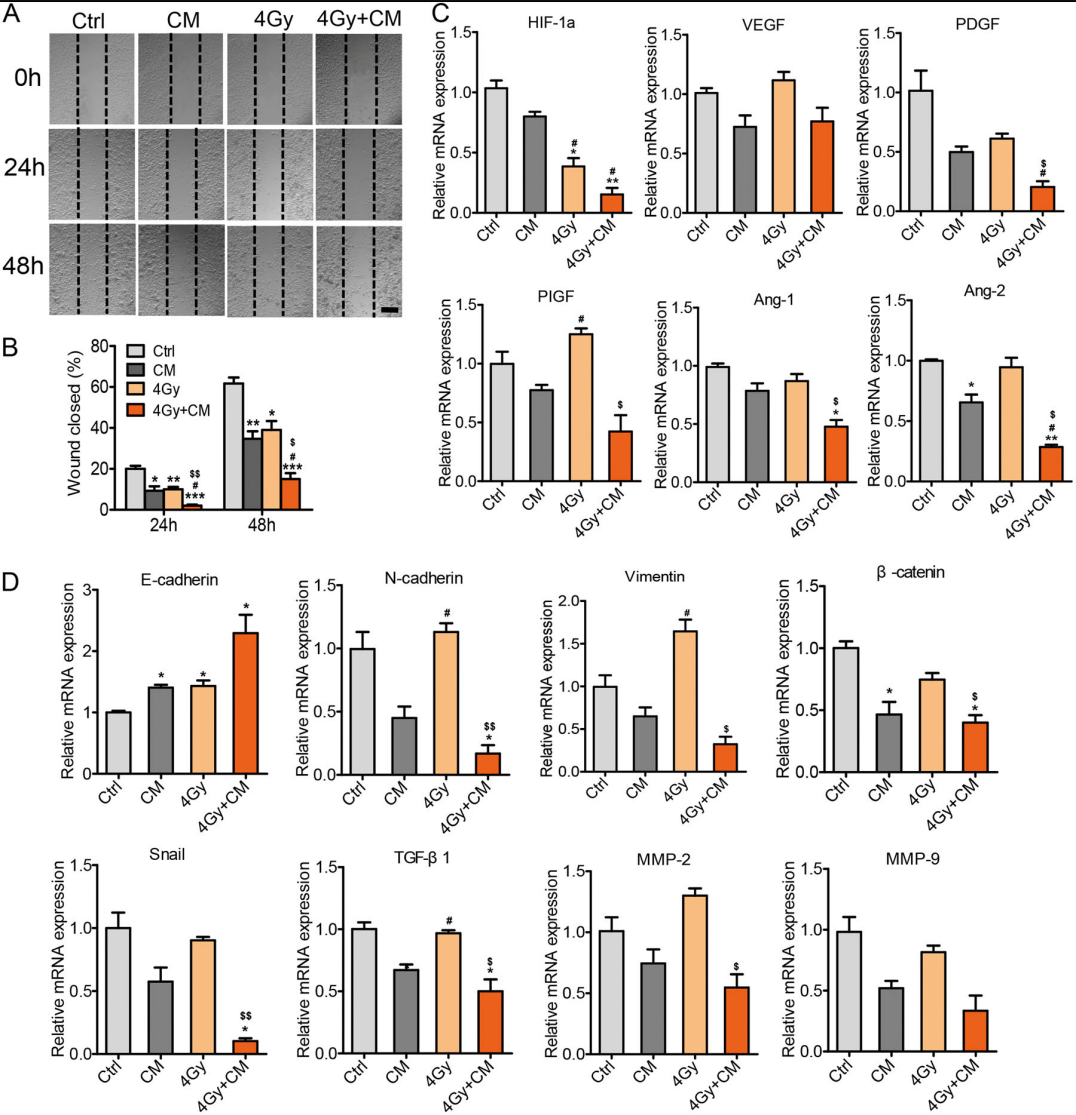
The effects of MSC-CM on MDA-MB-231 cell migration, metastasis and angiogenesis. (**a**) Representative photographs of wound-healing assays of MDA-MB-231 cells treated with MSC-CM and 4 Gy irradiation. The scale bar represents 100 μm. (**b**) Wound healing area per field was decreased after treatment with MSC-CM and MSC-CM+4 Gy. *P < 0.05 vs Ctrl, **P < 0.01 vs Ctrl, ***P < 0.001vs Ctrl, ^#^*P* < 0.05 vs MSC-CM, ^$^*P* < 0.05 vs 4 Gy, ^$$^*P* < 0.01 vs 4 Gy, n = 3. (**c**) Analysis of angiogenic factor expression in MDA-MB-231 cells in different groups. *P < 0.05 vs Ctrl, **P < 0.01 vs Ctrl, ^#^*P* < 0.05 vs MSC-CM, ^$^*P* < 0.05 vs 4 Gy, n = 3. (**d**) Expression of metastasis and EMT-related genes was determined by RT-PCR. All experiments were performed at least in triplicate, and the data are presented as the mean±SD. *P < 0.05 vs Ctrl, ^#^*P* < 0.05 vs MSC-CM, ^$^*P* < 0.05 vs 4 Gy, ^$$^*P* < 0.01 vs 4 Gy, n = 3. See also Table S1

Our data indicated that MDA-MB-231 cells treated with MSC-CM and radiation exhibited substantial inhibition of cell proliferation and Stat3 activation. Then, we examined the effects of MSC-CM on the migration, metastasis, and angiogenesis of cancer cells. Wound scratch assays were performed to test cell migration in different groups. After 48 h of MSC-CM, radiation or a combination of CM and radiation treatment, a significant difference was observed between the combination treatment and control groups. The results indicated that MSC-CM impaired tumour cells migration ability (Fig. 5a, b). As tumour cell migration is closely associated with EMT^37^, then we examined epithelial and mesenchymal biomarker expression. The RT-PCR results showed that treatment with CM and radiation decreased Vimentin and N-cadherin expression (mesenchymal markers) and increased E-cadherin expression (epithelial markers). Activated Stat3 regulates tumour invasion by regulating the gene transcription of matrix metalloproteinase 2 (MMP-2), MMP-9, TGF-β1 and β-catenin, which also decreased after treatment with MSC-CM (Fig. 5d). These results indicated that the inhibitory effect of MSC-CM in combination with radiotherapy on the tumour metastasis is probably through the EMT pathway.

Phosphorylated Stat3 was shown to bind to HIF-1α directly, and Stat3 has also been shown to enhance HIF-1α-mediated expression of VEGF^38^. Therefore, we examined tumour angiogenesis-related gene expression. The results indicated that MSC-CM maybe inhibit angiogenesis (Fig. 5c). All these data showed that the MSC-CM combination with radiotherapy could inhibit the migration, metastasis, and angiogenesis of breast cancer cells.

### DNA repair is inhibited by decreased Stat3

**Fig. 6:**
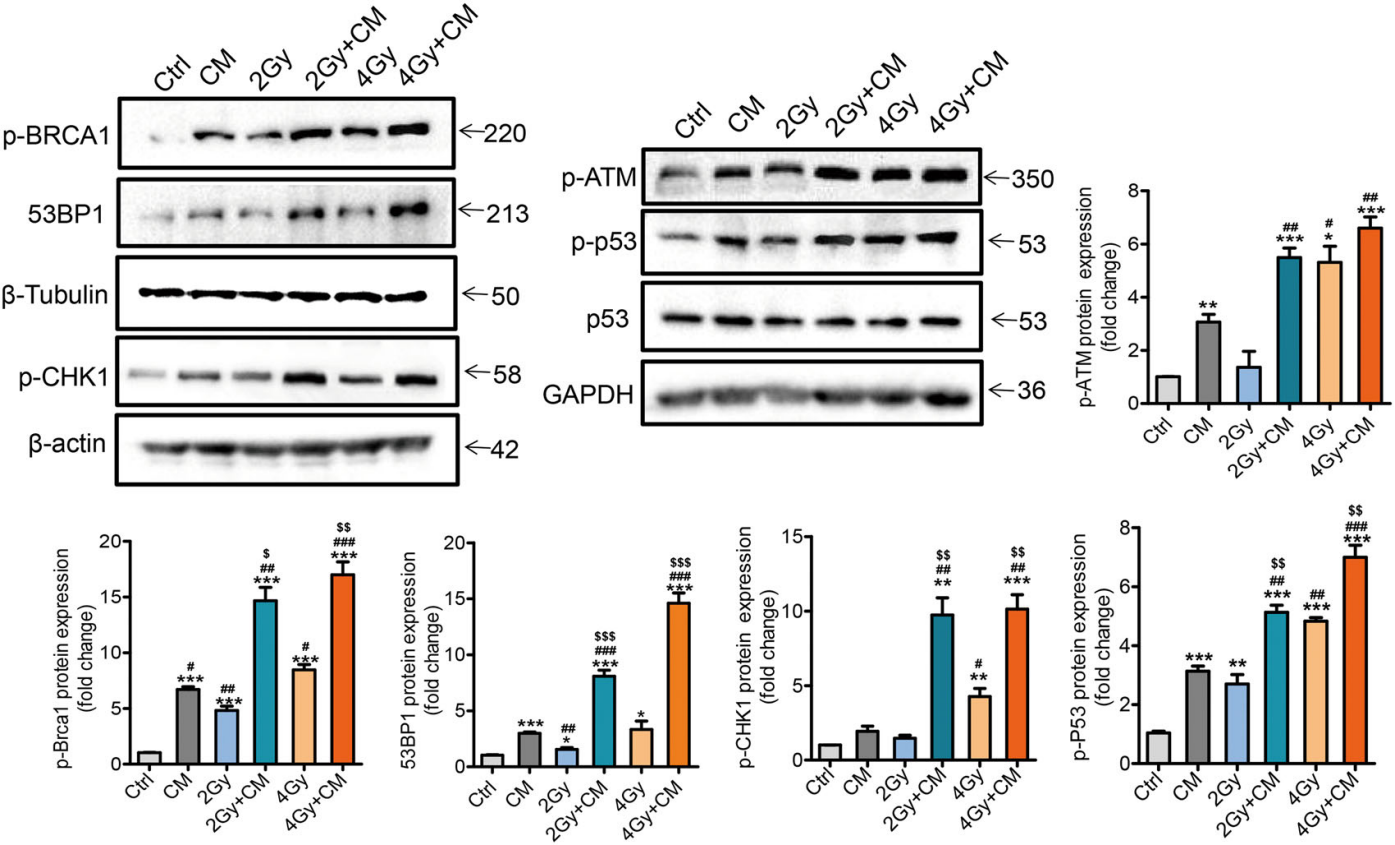
DNA repair is inhibited by Stat3 inhibition. MSC-CM and the MSC-CM combination with radiation increased p-BRCA1, 53BP1, p-ATM, p-p53 and p-CHK1expression. Cells were treated with MSC-CM or treated with ionizing radiation. Six hours after ionizing radiation, cells were harvested, and the cell lysates were analysed by immunoblotting using the indicated antibodies. *P < 0.05 vs Ctrl, **P < 0.01 vs Ctrl, ***P < 0.001 vs Ctrl, ^#^*P* < 0.05 vs MSC-CM, ^##^*P* < 0.01 vs MSC-CM, ^###^*P* < 0.001 vs MSC-CM, ^$$^ *P* < 0.01 vs 4 Gy, ^$$$^*P* < 0.001 vs 4 Gy, n = 3

We demonstrated that cells treated with MSC-CM exhibited strong down-regulation of Stat3 expression. As the Stat3 pathway has been shown to play an important role in the radiotherapy of cancers^39^ primarily through regulation of the radiation-induced DSBs^40^, we tested the possible impact of MSC-CM treatment on the regulation of DNA damage. Several factors involved in repair of DSBs were assessed, including BRCA1 (breast cancer 1, early onset), which is a well-known tumour suppressor with critical roles in DNA repair^41^; 53BP1, which is an important regulator of DSB repair protecting broken DNA ends from processing, a mechanism that is induced by BRCA1^42,43^; and ATM, which is the master regulator of the DNA damage response^44^. Our data showed that the protein expression levels of p-BRCA1, 53BP1, and p-ATM were increased after treatment with MSC-CM, especially in the combination group (Fig. 6). We assessed γH2AX and 53BP1 foci as indicators of DNA DSBs by immunostaining (Figure S2). According to regular statistical standard for analyzing DNA damage repair protein foci^45,46^, cells with >10 foci were counted as positive cells. As expected, dsDNA damage was significantly increased in the combination group, and this damage persisted significantly longer (24 h) compared with that of the control group. The effect on regulators of cell cycle arrest was also evaluated in our current study by assessing the two transducers of CHK1 (checkpoint kinase-1) in the ATM pathway^47^ and the central stress protein p53 in the DNA damage response^48,49^. Our results showed that the protein levels of p-p53 and p-CHK1 were increased in the combination groups. These results indicated that MSC-CM enhanced DNA damage after radiation, which increased the radiosensitivity of tumour cells.

### The MSC-CM combination with radiotherapy inhibits Stat3 signaling activation in MDA-MB-231 cells in vivo

**Fig. 7:**
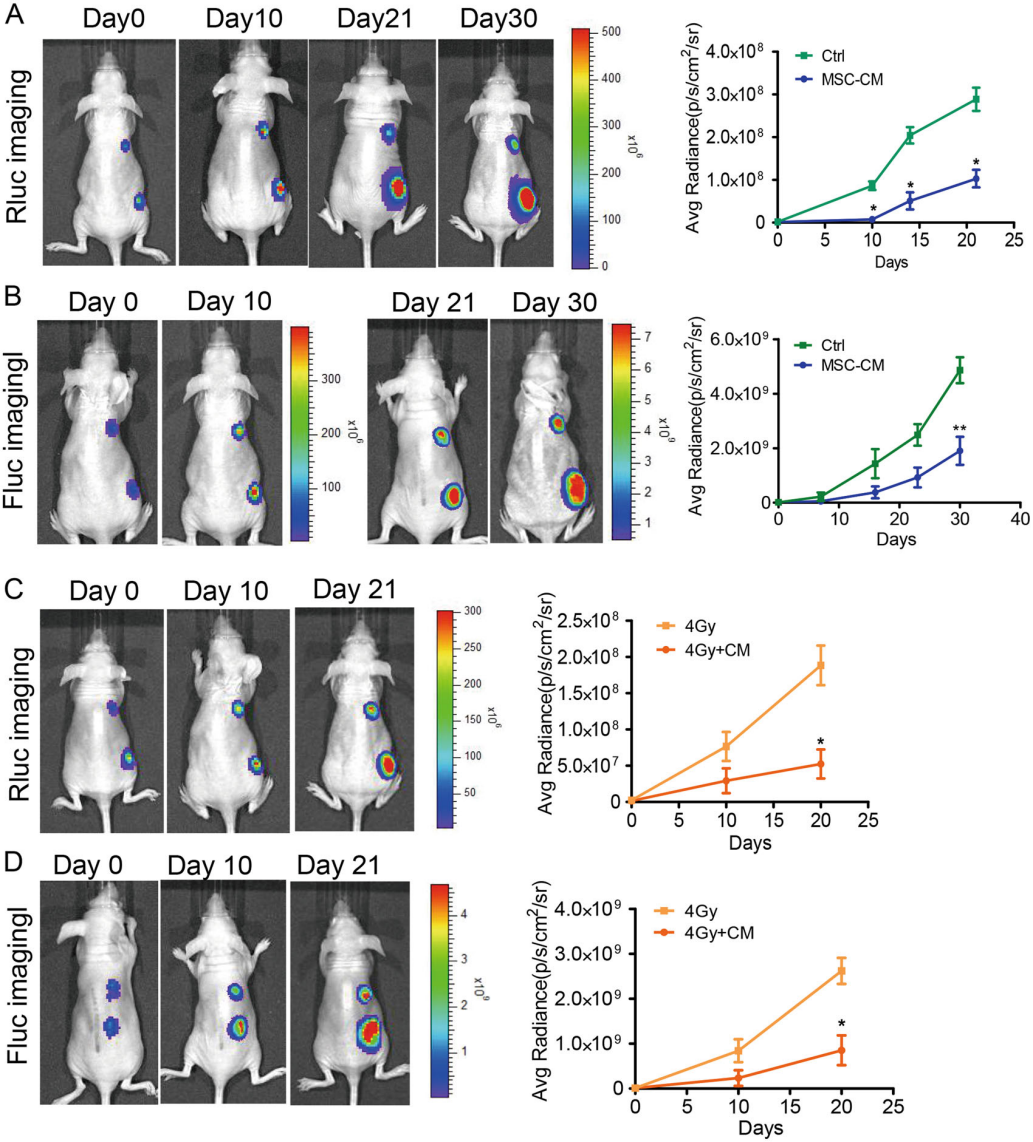
Inhibitory effect of MSC-CM on Stat3 signaling and tumour growth in vivo. (**a**) Renilla luciferase (Rluc) imaging of p-Stat3. Representative animals were injected with 2 × 10^6^ MDA-MB-231 (Fluc/GFP-pStat3/Rluc) cells into the upper and lower right side of the mouse body after treatment with MSC-CM and control medium for 48 h. Quantitative analysis of Rluc signals indicated decreased p-Stat3 level. *P < 0.05 vs Ctrl, n = 6. (**b**) Firefly luciferase (Fluc) imaging of tumour growth. Representative animals injected with 2 × 10^6^ MDA-MB-231 (Fluc/GFP-pStat3/Rluc) cells into the upper and lower right of the mouse body after treatment with MSC-CM and control medium for 48 h. Quantitative analysis of the Fluc signal. **P < 0.01 vs Ctrl, n = 6. (**c**) Renilla luciferase (Rluc) imaging of p-Stat3. Representative animals injected with 2 × 10^6^ MDA-MB-231 (Fluc/GFP-pStat3/Rluc) cells into the upper and lower right side of the mouse body after treatment with MSC-CM combination with radiation or radiation alone. Quantitative analysis of Rluc signal. *P < 0.05 vs 4 Gy, n = 6. (**d**) Firefly luciferase (Fluc) imaging of tumour growth. Representative animals injected with 2 × 10^6^ MDA-MB-231 (Fluc/GFP-pStat3/Rluc) cells into the upper and lower right side of the mouse body after treatment with MSC-CM combination with radiation or radiation alone. Quantitative analysis of Fluc signal. *P <  0.05 vs 4 Gy, n = 6

To determine whether the pre-treatment of MSC-CM combination with radiotherapy on cells could inhibit Stat3 activation in vivo, we subcutaneously implanted equal numbers of MDA-MB-231 cells (Fluc/GFP-pStat3/Rluc) that were treated with MSC-CM or control medium into the upper and lower right region of Nu/Nu Nude mice. Tumour cells pre-treated with MSC-CM combined with 4 Gy radiation or radiation alone were injected into the upper and lower right region of the mouse body. Bioluminescence imaging of Rluc was performed to assess Stat3 activation in the tumours. The imaging results demonstrated that the MSC-CM combination with radiotherapy pre-treatment could significantly inhibited the Stat3 signaling pathway in vivo (Fig. 7a, c).

**Fig. 8:**
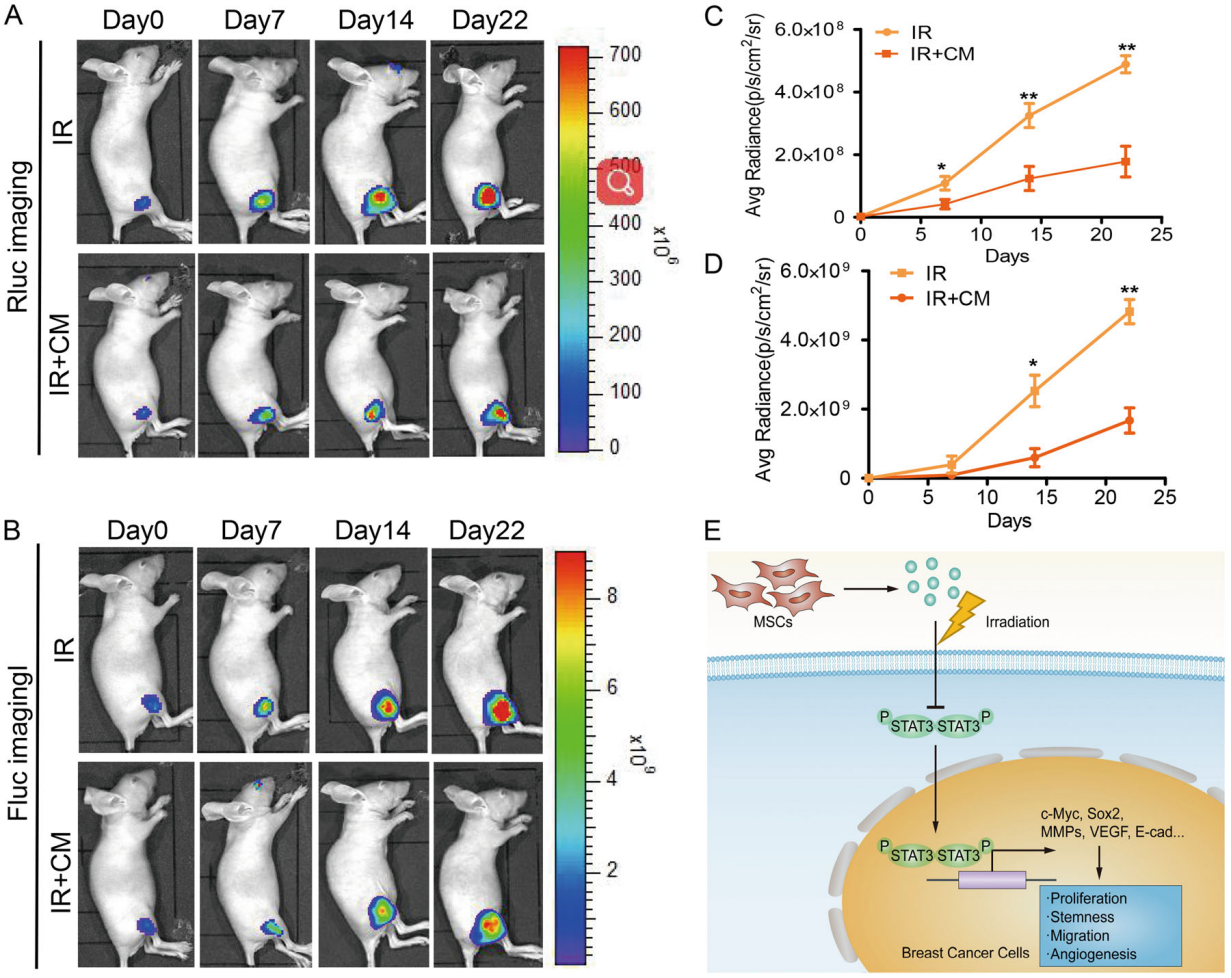
MSC-CM sensitizes MDA-MB-231 cells to irradiation in vivo. (**a**) Renilla luciferase (Rluc) imaging of p-Stat3. Representative animals were injected with 2 × 10^6^ MDA-MB-231 (Fluc/GFP-pStat3/Rluc) cells into the right hind leg first, and then received radiotherapy to the animal tumour with or without MSC-CM treatment. (**b**) Firefly luciferase (Fluc) imaging of tumour growth. Representative animals injected with 2 × 10^6^ MDA-MB-231 (Fluc/GFP-pStat3/Rluc) cells into the right hind leg first, and then received radiotherapy to the animal tumour with or without MSC-CM treatment. (**c**) Quantitative analysis of Rluc signals indicated decreased p-Stat3 level. *P < 0.05 vs IR, **P < 0.01 vs IR, n = 6. (**d**) Quantitative analysis of the Fluc signal. *P < 0.05 vs IR, **P < 0.01 vs IR, n = 6. (**e**) Proposed model for tumour-suppressive and radiosensitivity effects of the MSC-CM combination with irradiation. Some factors secreted by MSC cells could efficiently inhibit Stat3 activation in MDA-MB-231 cells. The Stat3 signalling pathway regulates the expression of c-Myc, Sox2, MMPs, VEGF, and E-cadherin and then is involved in cancer cell growth, stemness, invasion, and angiogenesis

To investigate the inhibition effect of MSC-CM combination with radiotherapy on Stat3 signaling pathway, we also performed another group of animal experiment in which cells were injected subcutaneously into the right hind leg of animal first, and then the effect of radiotherapy to the animal tumour with or without MSC-CM treatment for the animal was observed. The Rluc activity increased more slowly in the MSC-CM combination with radiotherapy group than the radiotherapy group (Fig. 8a, c).

### MSC-CM combined with radiotherapy significantly delays xenograft tumour growth

To confirm whether the down-regulation of Stat3 leads to tumour growth inhibition, we performed Fluc imaging to detect the tumour growth in vivo. Cells were pre-treated with MSC-CM, 4Gy radiation or MSC-CM combination with 4Gy radiation before injecting subcutaneously into the mice. From the data, we discovered that the signal intensity of cells treated with CM combined with radiation increased more slowly than the cells in the control group (Fig. 7b, d). These data demonstrated that MSC-CM weakened the tumour formation ability and inhibited tumour growth by repressing the Stat3 signaling pathway.

To further explore the inhibition effect of MSC-CM combination with radiotherapy on tumour growth, we injected cells subcutaneously into the right hind leg of animal first, and then observed the effect of radiotherapy to the animal tumour with or without MSC-CM treatment through Fluc imaging. The results demonstrated that the tumours grew more slowly in MSC-CM combination with radiotherapy group than the only radiotherapy group (Fig. 8b, d).

## Discussion

Radiotherapy is a critical therapeutic method for breast cancer. However, its therapeutic effect depends on the radioresistance of cancer cells. In this study, we report several meaningful findings. First, inhibitory growth of MDA-MB-231 breast cancer cells treated by MSC-CM was observed. Second, MSCs could enhance the therapeutic effect of radiotherapy by inhibiting the Stat3 signal pathway. To our knowledge, no other reports have indicated the molecular mechanisms underlying MSC-mediated radiosensitivity in MDA-MB-231 cells. In addition, given that MSCs have not only been investigated for the treatment of cancers^50,51^ but also for the management of cancer radiosensitivity^3^, it is noteworthy that we uncovered the interplay between the factors secreted by MSCs and the Stat3 pathway which influences cellular radiosensitivity, and this will help develop novel Stat3-targeting therapies.

Some reports have shown the MSC-mediated suppression of cancer cell proliferation^4,52,53^, indicating that MSCs inhibit the growth and promote the apoptosis of cancer cells. In addition, one report on breast cancer cells further showed that MSCs significantly down-regulated the expression of VEGF in tumour cells, which led to inhibition of angiogenesis in vitro and in vivo. Moreover, other studies showed that MSCs enhanced the radiotherapy effect on cancers likely through inhibition of tumour cell proliferation and enhancement cancer cell apoptosis^3^. Thus, inhibitory cell growth, low colony-forming efficiency and cell apoptosis were observed in our data (Fig. 2). However, the molecular mechanism of MSC-induced tumour suppression remains poorly understood. In the current study, we demonstrated that MSC-CM could inhibit MDA-MB-231 cell proliferation, migration, and angiogenesis and promote DNA damage, likely by inhibiting the Stat3 pathway as shown by bioluminescence imaging technology.

Stat3 is a member of the Stat protein family, in response to cytokines and growth factors. Stat3 becomes activated after phosphorylation of tyrosine residue (Y705)^54^. Recent studies suggest that Stat3 plays a central role in development of cancer radioresistance^55,56^. Stat3 has been shown to enhance expression of regulatory factors related to cancer cell proliferation, metastasis, angiogenesis, and resistance to apoptosis-related genes^57^. Therefore, inhibition of Stat3 may be a promising therapeutic strategy for the management of cancer radioresistance^58,59^. Indeed, as shown in Fig. 4a, c in our study, the phosphorylated Stat3 expression levels were decreased in the combination treatment groups. These results suggest that radiosensitivity of MDA-MB-231 cells can be regulated by the Stat3 pathway, a hypothesis supported by evidence demonstrating that inhibition of the Stat3 pathway sensitises cancer cells to radiation^56,60–62^.

Regarding the interplay between the radiosensitivity effect of MSCs on cancer cells and the inhibition of the Stat3 signaling pathway, to our knowledge, no report has demonstrated that MSC-CM inactivates the Stat3 pathway after radiation in MDA-MB-231 cells. To test our hypothesis, in addition to evaluating the Stat3 activation via bioluminescence imaging and cell-based ELISA, we also determined ALDH expression and the Mammosphere formation ability which are widely accepted to identify the CSCs. Numerous studies have found that Stat3 activation is involved in regulating radioresistance of cancers, which could be due to its central roles in CSC maintenance^61,63,64^. CSCs were reported to induce tumour growth, radioresistance, metastasis and angiogenesis in breast cancer^65^. Therefore, we found that the migration, invasion and angiogenesis abilities of tumour cells were weakened in combination groups (Fig. 5a–d).

Findings of this study indicate that suppressing the Stat3 signaling pathway combined with traditional radiotherapy could be a promising novel approach for overcoming radioresistance in MDA-MB-231 breast cancer cells. However, an inherent limitation of our study is that the factors in MSC-CM mediating the inactivation of Stat3 were unclear. A further study that specifically elucidates how MSC-CM results in the inhibition of Stat3 and thus regulates the radiosensitivity of cancer cells will be performed in the future.

In conclusion, we demonstrated that MSC-CM is a potent tumour suppressor of MDA-MB-231 cells by inhibiting Stat3 activation and down-regulating its downstream gene expression. Our data indicate that Stat3 could be a potential target and MSCs could provide a basis for a promising therapeutic strategy for radiotherapy of breast cancer.

## Materials and methods

### Reagents

Heat-inactivated fetal bovine serum (FBS) and cell culture medium DMEM, DMEM/F12 were purchased from Gibco (Invitrogen China Limited, China). A real-time PCR (RT-PCR) kit was purchased from TransGen Biotech (Beijing, China). D-Luciferin was purchased from Biosynth International (Naperville, USA). Coelenterazine was purchased from NanoLight Technology (Pinetop, AZ, USA). Antibodies against p-BRCA1 (Ser1524), 53BP1, p-ATM (Ser1981), p-p53 (Ser15), p53, p-CHK1 (Ser317), p-Stat3 (Tyr705), Stat3 were purchased from Abcam (Cambridge, MA, USA). Antibodies against β-actin, β-tubulin, and GADPH were obtained from ZSGB-Bio (China).

### Cell culture

The human breast cancer cell line MDA-MB-231 was purchased from ATCC (Manassas, VA, USA), and human umbilical cord MSCs (hUC-MSCs) were isolated and cultured as described previously^66^. MDA-MB-231 cells were grown in DMEM medium supplemented with 10% foetal bovine serum (FBS), 1% penicillin streptomycin solution (Gibco, Rockville, MD), and 1% MEM non-essential amino acid solution (Gibco). The culture medium of hUC-MSCs was DMEM/F12 (Gibco), 10% FBS, penicillin (100 units/ml), streptomycin (100 mg/ml)(Gibco). For imaging, MDA-MB-231 cells were conducted with double imaging reporter genes. The reporter construct carried firefly luciferase (Fluc) and enhanced green fluorescence protein (eGFP) drived by an ubiquitin promoter, Renilla luciferase (Rluc) reporter gene drived by a 7-repeat of Stat3-recognition sites (enhancer) and a small TA promoter in response to phosphorylated Stat3.

### Collection of conditioned medium

The hUC-MSC cells and MDA-MB-231 cells (purchased from ATCC) were cultured to 40% confluence in the previously described culture media, and the medium for hUC-MSCs cells was replaced with 7 ml DMEM/F12 medium. The medium for MDA-MB-231 cells was replaced with 7 ml DMEM. After 2 days, the supernatants were collected and stored at –80 °C.

### Morphological observation

2.5 × 10^5^ of MDA-MB-231 cells were seeded into six-well plate. The medium was replaced with hUC-MSC-CM and 231-CM mixed with the same amount of DMEM medium (containing 10% FBS) on the next day.

### Cell proliferation and viability assays

MDA-MB-231 cells (3 × 10^4^) were seeded in 12-well plates. The medium was replaced with different CMs and mixed with DMEM medium (containing 10% FBS) on the next day. Cells in plate were counted in three days. Cell viability was determined by Trypan blue dye exclusion assays.

### Bioluminescence imaging

Bioluminescence imaging of the fate of transplanted cells in living mice was performed for 30 days using an IVIS cooled CCD optical Imaging System (Xenogen Corporation, Hopkinton, MA). Fluc imaging of MDA-MB-231 cells was used to assess tumour progression, and the expression of phosphorylated-Stat3 was determined by using Rluc imaging. Fluc imaging was performed with D-Luciferin (150 mg/kg; Biosynth International, Naperville, IL, USA), the reactive substrate that was intraperitoneally injected into mice. Coelenterazine (2.5 mg/kg; NanoLight Technology, Pinetop, AZ, USA) was used to evaluate Rluc expression^51,67^.

### Cell irradiation

A Cs-137 (Gammacell-40) irradiator was purchased from Atomic Energy Co. (Atomic Energy of Canadian Inc., Mississauga, ON, Canada). Cell or animal samples were placed in the centre of the irradiation chamber and exposed to the radiation at a dose rate of 1.02 Gy/min.

### Real-time PCR

To assess the mRNA expression levels of the genes, total RNA was extracted from MDA-MB-231 cells treated with TRIzol reagent according to instructions. 2 μg of total RNA was reverse transcribed using a Reverse Transcription Kit (TIANGEN Biotech), and real-time PCR was performed with the Opticon® System (Bio-Rad) using a TransStart Green qPCR Super Mix Kit (TransGen Biotech). The 2^–ΔΔct^ method was used to assess the relative mRNA expression.Primers are listed Supplemental Table 1.

### Western blot analysis

To determine the protein expression, western blotting was performed. MDA-MB-231 cells were lysed on ice in lysis buffer (Cwbiotech, Beijing, China) including protease inhibitor cocktail. The total protein was quantified with BCA Protein Assay Kit as described previously [21]. Antibodies, anti-p-BRCA1 (Ser1524), anti-53BP1, anti-p-ATM(Ser1981), anti-p-p53(Ser15), anti-p53, anti-p-CHK1(Ser317), anti-p-Stat3(Tyr 705), anti- Stat3, anti-β-actin, anti-β-tubulin and anti-GADPH.

### Cell-based ELISA

The activated Stat3 expression in MDA-MB-231 cells was detected by Cell-Based Colorimetric ELISA Kit (ImmunoWay, USA). After treatment with formaldehyde, cells were incubated with phospho-Stat3 (Try705) and total Stat3 antibodies and then the secondary HRP-conjugated antibody. The Stat3 phosphorylation levels were normalised by both the levels of total Stat3 protein and total cell number in each well^68^.

### Flow cytometric apoptosis assay

Apoptosis was measured by fluorescence-activated cell sorting (FACS) using the Annexin V-FITC Apoptosis Detection Kit. In brief, cells plated in 6-well plates were treated with MSC-CM for 48 h or in combination with radiotherapy. After 48 h of radiation, the cells were harvested for cell apoptosis assay and analysed by flow cytometry.

### Colony formation assay

MDA-MB-231 cells were trypsinised, suspended in a single-cell suspension and plated into 6-well plates (800 cells/well). Then the next day, the medium in control group was replaced with 231-CM and DMEM (including 10% FBS), and the experimental group was MSC-CM and DMEM (including 10% FBS) at a ratio of 1:1. Cells were treated with the indicated doses of ^137^Cs–radiation (1.02 Gy/min). After 10 days culture, the cells were treated with crystal violet. Colonies containing 50 cells or more were counted as described previously^69,70^.

### Cell-cycle analysis

After treatment with MSC-CM or a combination with radiotherapy, cells were harvested and washed with PBS. Then cells were treated with methanol and resuspended in PI-staining buffer (50 μl/ml PI, RNase A) for 15 min at 37 °C Fluorescence intensity was analyzed by flow cytometer.

### Wound-healing assay

MDA-MB-231 cells (2 × 10^5^) were plated in six-well plate. Then the medium was replaced with control medium or a mixture of MSC-CM with the same amount of DMEM (supplemented with 10% FBS). Then, cells were exposed to 4 Gy radiation. For scratch wound healing assay, cells were cultured in serum-free medium for 24 h and three separate wounds were made with sterile pipette tips (Corning). The percentage of wound closing was assessed using the following formula:

[1–(final area/initial area)] × 100%.

### Immunofluorescence staining

A total of 1 × 10^4^ cells were plated into each well of 12-well plates. After treatment with control medium or MSC-CM for 48 h, cells were exposed to 4 Gy radiation. The cells in four groups (Ctrl, CM, 4Gy, 4Gy+CM) were fixed with 4% paraformaldehyde (Sigma) for 15 min. Cells were first incubated with primary antibody, mouse anti-phospho-Histone H2AX (Ser139), rabbit anti-53BP1, followed by incubation with Cy3 goat anti-mouse IgG and goat anti-rabbit secondary antibodies. And then counter stained with DAPI. Images were acquired using an AMG EVOS microscope.

### Mammosphere assay

Cells were grown in serum-free, growth factor-enriched conditions in low-attachment plates to generate mammospheres^71–73^. MDA-MB-231 cells were treated with control and MSC-CM for 48 h and then exposed to 4 Gy radiation. Then the cells were seeded into ultralow-attachment plates and cultured in serum-free DMEM medium containing basic fibroblast growth factor (bFGF), human epidermal growth factor (hEGF), and B27 (Invitrogen). The number of formed mammospheres was determined under an inverted microscope.

### Aldefluor-positivity assay

The Aldefluor kit (Stem Cell Technologies) was used to assess ALDH activity with flow cytometry according to previous report^71^. Cells were suspended in ALDEFLUOR assay buffer containing ALDH substrate (Bodipy-Aminoacetaldehyde) and incubated at 37 °C for 45 min. As a negative control, 15 μM diethylaminobenzaldehyde (DEAB) was used. The fluorescent ALDH1-expressing cells were analyzed with flow cytometer.

### In vivo xenograft studies

A group of 8–12-week-old female Nu/Nu nude mice were housed under standard laboratory conditions. A group of mice (n = 6 mice/group) were injected with 2 × 10^6^ MDA-MB-231 (Fluc/GFP-pStat3/Rluc) cells into the upper and lower right region of the mouse body (day 0) after treatment with MSC-CM or control medium for 48 h^36^. Equal numbers of MDA-MB-231 (Fluc/GFP-pStat3/Rluc) cells after treatment with MSC-CM combined with radiation or radiation alone were injected into the upper and lower right of mice body to detect the tumour growth and the activated Stat3 expression level by imaging.

Another group of mice were injected with 2 × 10^6^ MDA-MB-231 (Fluc/GFP-pStat3/Rluc) cells into the right hind leg of animal first (day 0), five days later, when the tumors were palpable, the mice were randomly divided into two groups (6 mice per group): a radiation group, which received γ-radiation radiotherapy of tumors in the legs twice weekly (2 Gy) for 2 weeks, and a combination group (CM + radiotherapy) treated with MSC-CM every two days for 2 weeks. For radiation treatment, mice were first anaesthetized and then placed onto a specially designed lead plate so as to radiate locally the tumors. The activated Stat3 expression level and tumour growth were detected by Rluc and Fluc imaging. All experimental procedures and protocols were conducted according to the guidelines of our local animal care and use committee.

### Statistical analysis

All data were analyzed using GraphPad Prism 5.0 software. Data were analyzed by two-tailed Student’s t test or Two-way ANOVA. A *P* value less than 0.05 was considered statistically significant. Data were shown of three independent experiments.

## Electronic supplementary material

Supplementary Information
