# Supplementary material for: MSCs inhibit tumor progression and enhance radiosensitivity of breast cancer cells by down-regulating Stat3 signaling pathway
Source: Cell Death Dis. 2018 Oct 8;9(10):1026. doi: 10.1038/s41419-018-0949-3 (PMC6175943; doi:10.1038/s41419-018-0949-3)
Supplement: Supplementary file 1 — Supplementary Information [file 41419_2018_949_MOESM1_ESM.docx]

**Supplementary Information:**

**MSCs inhibit tumour progression and enhance radiosensitivity of breast cancer cells by down-regulating Stat3 signaling pathway**

**Supplementary Table S1**

**Supplementary Figures1-4 legends**

**Table S1. Primer sequences used for real-time RT-PCR**

| Gene | Forward primer (5’→3’) | Reverse primer (5’→3’) |
| --- | --- | --- |
| Nanog | CCCCTCCTCCCATCCCTC | GCTCCAACCATACTCCACCC |
| Oct4 | GTATTCAGCCAAACGACCATC | GCTTCCTCCACCCACTTCT |
| Sox2 | GGTTACCTCTTCCTCCCACTCC | CCTCCCATTTCCCTCGTTT |
| Bcl-xl | GATCCCCATGGCAGCAGTAAAGCAAG | CCCCATCCCGGAAGAGTTCATTCACT |
| Stat3 | GGCATTCGGGAAGTATTGTCG | GGTAGGCGCCTCAGTCGTATC |
| c-Myc | AATGAAAAGGCCCCCAAGGTAGTTATCC | GTCGTTTCCGCAACAAGTCCTCTTC |
| CyclinD1 | AGCTCCTGTGCTGCGAAGTGGAAAC | AGTGTTCAATGAAATCGTGCG GGGT |
| Ang-1 | CAGCGCCGAAGTCCAGAAAAC | CACATGTTCCAGATGTTGAAG |
| Ang-2 | GACTGGGAAGGCAACGAG | CTGAGAGCATCTGGGAACA |
| HIF-1α | TGCTTGGTGCTGATTTGTGA | GGTCAGATGATCAGAGTCCA |
| PIGF | CTTCTGAGTCGCTGTAGTGG | TCCTTTCTGCCTTTGTCG |
| PDGF | GCCGAGTTGGACCTGAACAT | TCTTGCACTCGGCGATCAT |
| VEGF | CTACCTCCACCATGCCAAGT | GCAGTAGCTGCGCTGATAGA |
| N-cadherin | ATGTGCCGGATAGCGGGAGC | TACACCGTGCCGTCCTCGTC |
| Vimentin | TCTCTGAGGCTGCCAACCG | CGAAGGTGACGAGCCATTTCC |
| Snail | CACTATGCCGCGCTCTTTC | GGTCGTAGGGCTGCTGGAA |
| E-cadherin | GAAGGTGACAGAGCCTCTGGAT | GATCGGTTACCGTGATCAAAATC |
| TGF-β1 | GGCCTTTCCTGCTTCTCATGG | CCTTGCTGTACTGCGTGTCC |
| MMP-2 | TCTCCTGACATTGACCTTGGC | CAAGGTGCTGGCTGAGTAGATC |
| MMP-9 | TTGACAGCGACAAGAAGTGG | GCCATTCACGTCGTCCTTAT |
| GAPDH | GCACAGTCAAGGCCGAGAAT | GCCTTCTCCATGGTGGTGAA |
| p53 | GCCCAACAACACCAGCTCCT | CCTGGGCATCCTTGAGTTCC |


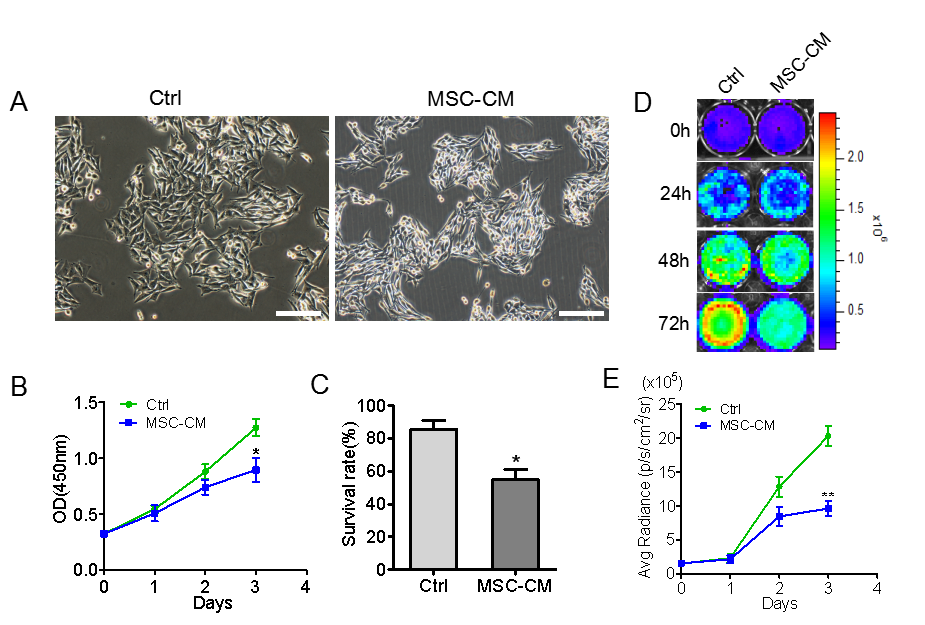


**Figure S1. Effect of MSC-CM on the proliferation of MDA-MB-231 cells.**

1. Microscopy showed a morphological change in cells treated with MSC-CM. The scale bar represents 100 μm.
2. Growth curves from the CCK-8 assays revealed a decreased proliferation rate of cells treated by MSC-CM. **P*<0.05 vs Ctrl, n=3.

(C) Trypan blue cell viability assays showed a low survival rate in the MSC-CM group. **P*<0.05 vs Ctrl, n=3.

(D) Fluc imaging showed decreasing bioluminescence signals at 48 and 72 h after cells were treated with MSC-CM.

(E) Quantitative analysis of Fluc signals. Bioluminescence activity showed the suppressed growth in the MSC-CM-treated group. ***P*<0.01 vs Ctrl, n=3.


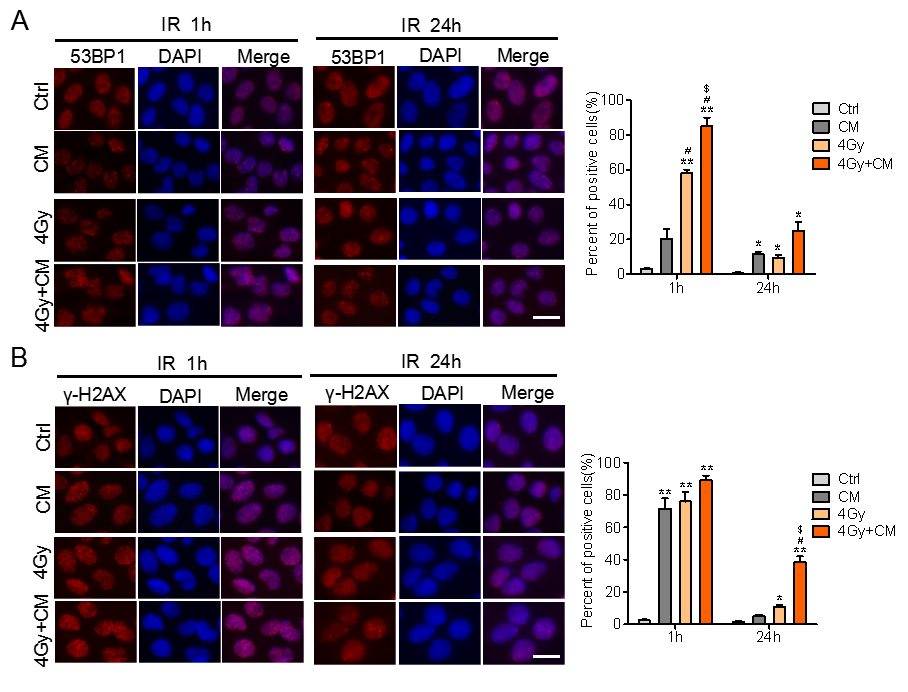


**Figure S2. Immunofluorescence staining of phosphorylated H2AX and 53BP1 foci.**

(A) After treatment with MSC-CM and exposure to 4 Gy irradiation, cells were stained with 53BP1 antibody and detected using a Cy3 secondary antibody.

(B) After treatment with MSC-CM and exposure to 4 Gy irradiation, cells were stained with phospho-histone H2AX (Ser139) antibody and detected using a Cy3 secondary antibody. Cells with >10 foci were counted as positive cells. **P*<0.05 vs Ctrl, ***P*<0.01 vs Ctrl, ^#^*P*<0.05 vs MSC-CM, ^$^ *P*<0.05 vs 4 Gy, n=3.


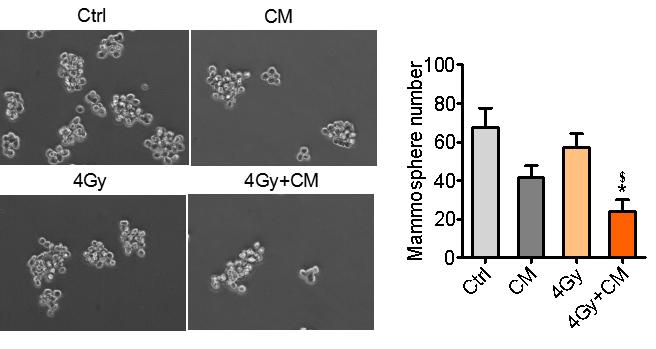


**Figure S3.** **Mammosphere formation of MDA-MB-231 cells.**

Cells after treatment with MSC-CM for 48 h and exposure to radiation were plated in low-density suspension cultures for sphere formation. Experiments were performed in triplicate. Data are presented as the mean±SD. **P*<0.05 vs Ctrl, ^$^ *P*<0.05 vs 4 Gy, n=3.


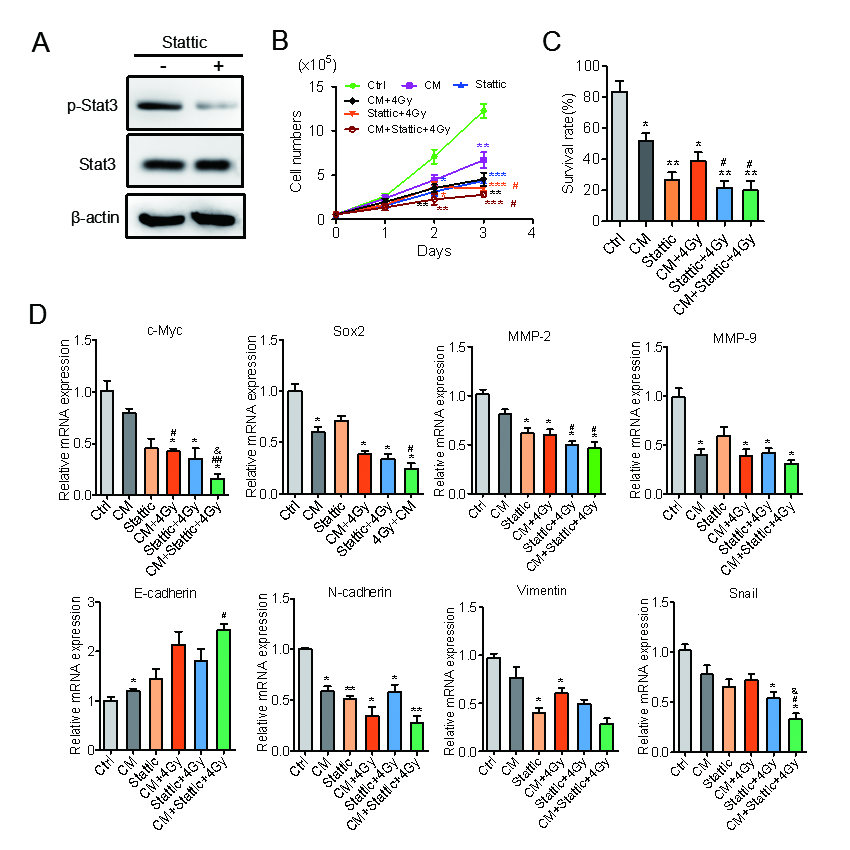
**Figure S4. Effect of MSC-CM on cancer cells after inhibited the Stat3 signaling pathway.**

(A) After treatment with 5uM Stattic for 24h, cells were harvested, and the cell lysates were analysed by immunoblotting using the indicated antibodies.

(B) Growth curves made by counting cell numbers every day revealed a decreased proliferation rate of cells treated by MSC-CM and the MSC-CM combination with 4 Gy radiation after Stattic administrated. *P<0.05 vs Ctrl, **P<0.01 vs Ctrl, ***P<0.001 vs Ctrl, ^#^*P*<0.05 vs MSC-CM, n=3.

(C) Trypan blue cell viability assays showed a low survival rate. *P<0.05 vs Ctrl, **P<0.01 compared to Ctrl, ^#^*P*<0.05 vs MSC-CM, n=3.

(D) Real time PCR analysis of genes expression. Stemness, metastasis and epithelial-mesenchymal transition were inhibited in MSC-CM combination with radiation group after treated with Stattic. **P*<0.05 vs Ctrl, ***P*<0.01 vs Ctrl, ^#^*P*<0.05 vs MSC-CM, ^##^*P*<0.01 vs MSC-CM, ^&^*P*<0.05 vs CM+4Gy, n=3.
